# Multi-method laboratory user evaluation of an actionable clinical performance information system: Implications for usability and patient safety

**DOI:** 10.1016/j.jbi.2017.11.008

**Published:** 2018-01

**Authors:** Benjamin Brown, Panos Balatsoukas, Richard Williams, Matthew Sperrin, Iain Buchan

**Affiliations:** a Health e-Research Centre, Farr Institute of Health Informatics Research, Centre for Health Informatics, University of Manchester, Manchester, UK; b NIHR Patient Safety Translational Research Centre Greater Manchester, University of Manchester, Manchester, UK

**Keywords:** A&F, audit and feedback, CDS, clinical decision support, e-A&F, electronic audit and feedback, Clinical audit, Medical audit, Clinical quality management, Clinical quality improvement, Clinical governance, User interface design, Clinical decision support

## Abstract

**Introduction:**

Electronic audit and feedback (e-A&F) systems are used worldwide for care quality improvement. They measure health professionals’ performance against clinical guidelines, and some systems suggest improvement actions. However, little is known about optimal interface designs for e-A&F, in particular how to present suggested actions for improvement. We developed a novel theory-informed system for primary care (the Performance Improvement plaN GeneratoR; PINGR) that covers the four principal interface components: clinical performance summaries; patient lists; detailed patient-level information; and suggested actions. As far as we are aware, this is the first report of an e-A&F system with all four interface components.

**Objectives:**

(1) Use a combination of quantitative and qualitative methods to evaluate the usability of PINGR with target end-users; (2) refine existing design recommendations for e-A&F systems; (3) determine the implications of these recommendations for patient safety.

**Methods:**

We recruited seven primary care physicians to perform seven tasks with PINGR, during which we measured on-screen behaviour and eye movements. Participants subsequently completed usability questionnaires, and were interviewed in-depth. Data were integrated to: gain a more complete understanding of usability issues; enhance and explain each other’s findings; and triangulate results to increase validity.

**Results:**

Participants committed a median of 10 errors (range 8–21) when using PINGR’s interface, and completed a median of five out of seven tasks (range 4–7). Errors violated six usability heuristics: clear response options; perceptual grouping and data relationships; representational formats; unambiguous description; visually distinct screens for confusable items; and workflow integration. Eye movement analysis revealed the integration of components largely supported effective user workflow, although the modular design of clinical performance summaries unnecessarily increased cognitive load. Interviews and questionnaires revealed PINGR is user-friendly, and that improved information prioritisation could further promote useful user action.

**Conclusions:**

Comparing our results with the wider usability literature we refine a previously published set of interface design recommendations for e-A&F. The implications for patient safety are significant regarding: user engagement; actionability; and information prioritisation. Our results also support adopting multi-method approaches in usability studies to maximise issue discovery and the credibility of findings.

## Highlights

• Explains the design rationale for a novel electronic audit and feedback system.
• Identifies usability issues through a multi-method laboratory user evaluation.
• Uses findings to inform the evidence-based user-centred design of computerised audit and feedback.
• Determines their implications for patient safety.

## Graphical abstract

**Figure:**
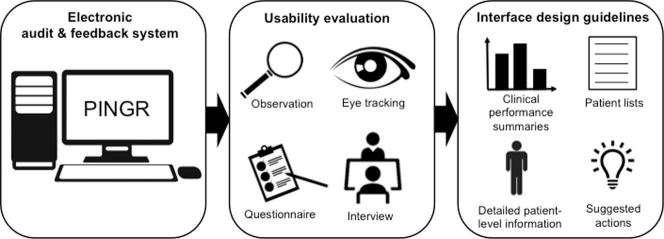

## 1 Introduction

Quality measurement is central to improvement strategies [1]. It identifies where action is needed and monitors the effects of improvement efforts [1]. In health care, this measurement is usually set in the context of ‘audit and feedback’ (A&F) or ‘clinical performance feedback’, where compliance with clinical standards or patient outcomes is the common metric [2]. Clinical performance is primarily fed back as ‘quality indicators’, ‘performance measures’, or similar quantities [2]. Electronic audit and feedback (e-A&F) systems communicate this information to health professionals mostly through interactive browser-based portals or desktop applications [3]. They are in use throughout the world, described variously as dashboards, benchmarking tools, scorecards etc [3].

Core to e-A&F systems is the presentation of quality indicators, which may be supplemented by the following components: patient lists; detailed patient-level information; and suggested actions [3]. Despite the potential importance of these components for actionable data interpretation [4], relatively little is known about designing usable interfaces for e-A&F to optimise user interaction and reduce errors during decision making [3]. In particular, existing evidence regarding e-A&F usability has been limited to systems without key interface components (e.g. suggested actions), and has largely ignored how interface design can affect user interaction when interpreting clinical performance data [3]. Evidence from the health informatics literature demonstrates that the design of information systems without regard for usability can increase technology-induced errors [5]. In the case of e-A&F systems such errors may have adverse consequences for patient safety by reducing the system’s effectiveness to improve health care outcomes [4]. Therefore poorly designed e-A&F interfaces may result in misinterpretation or ignorance of important information, which could ultimately lead to failings in care quality and efficiency (e.g. [6]).

We have previously reported a usability inspection evaluation of an e-A&F system for primary care – the Performance Improvement plaN GeneratoR; PINGR [3]. PINGR is currently unique among published e-A&F systems in that it possesses all key interface components: clinical performance summaries (i.e. quality indicators); patient lists; detailed patient-level information; and suggested actions [3]. Its design employs existing evidence and theory regarding effective A&F, and is intended to be generic so it can host quality indicators from a range of clinical areas. Consequently, usability findings from PINGR provide valuable insights into how to best design interfaces for e-A&F systems, and the findings may generalise to other settings such as secondary care. The results of PINGR’s usability inspection study enabled us to create a set of generic interface design recommendations for e-A&F systems, covering each of their interface components and how they can be integrated [3]. The study also represented the first step in an iterative approach to optimise PINGR prior to deployment in routine clinical practice [5], [7].

The present study extends usability testing to target end-users (primary care clinicians) as planned in PINGR’s development framework [5]. We seek to understand how the interface helps or hinders user interaction across a range of information interpretation and decision-making scenarios in clinical quality improvement. To achieve this we used a multi-method study design, collecting and analysing multiple types of qualitative and quantitative data [8]. Multi-method studies have been extensively used in both the natural and social sciences to combine different types of qualitative and quantitative data, such as self-administered questionnaires, semi-structured interviews, and ethnographic observations [9]. Common uses for integrating these different data include but are not limited to: gaining a more comprehensive account of the phenomenon of interest (*completeness*); augmenting or building on each others’ results (*enhancement*); explaining findings from another dataset (*explanation)*; and corroborating or disconfirming each others’ findings in order to increase validity (*triangulation*) [10]. Multi-method approaches are particularly suitable for usability studies in clinical informatics given the increasing complexity of modern information systems [11]. They have been found to more comprehensively uncover usability issues [12], and address different aspects of usability through triangulation and complementarity [13], than either of their constituent methods alone. However, challenges remain with regard to how to most efficiently and effectively synthesise these different data sources [14]. Consequently, the originality of this work lies in studying not only how primary care clinicians interact with e-A&F systems, but also how laboratory-based multi-method usability evaluations may be conducted.

### 1.1 Aim and objectives

The aim was to understand, through usability testing with end-users and theory-based abstraction, how the design of clinical e-A&F interfaces could facilitate improvements in patient safety.

The objectives were to:

1. test the usability of PINGR in terms of efficiency, errors, satisfaction, and utility, using a multi-method approach, combining data from observations of on-screen and visual search behaviour during task performance, post-test user satisfaction questionnaires, and in-depth interviews;
2. use these findings to extend and refine our previous set of interface design recommendations for e-A&F systems [3] in relation to their main interface components (clinical performance summaries; patient lists; detailed patient-level information; and suggested actions), whilst comparing them to the wider usability literature; and
3. determine the implications of these interface design recommendations for patient safety by drawing on evidence regarding clinical audit and feedback implementation.

## 2 Materials and methods

### 2.1 The evaluated system: PINGR

**Fig. 1:**
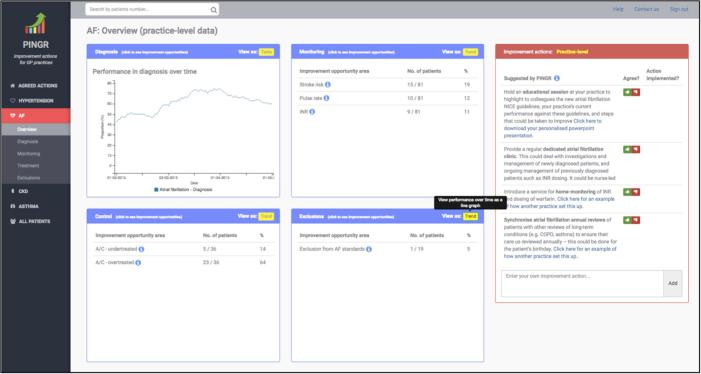

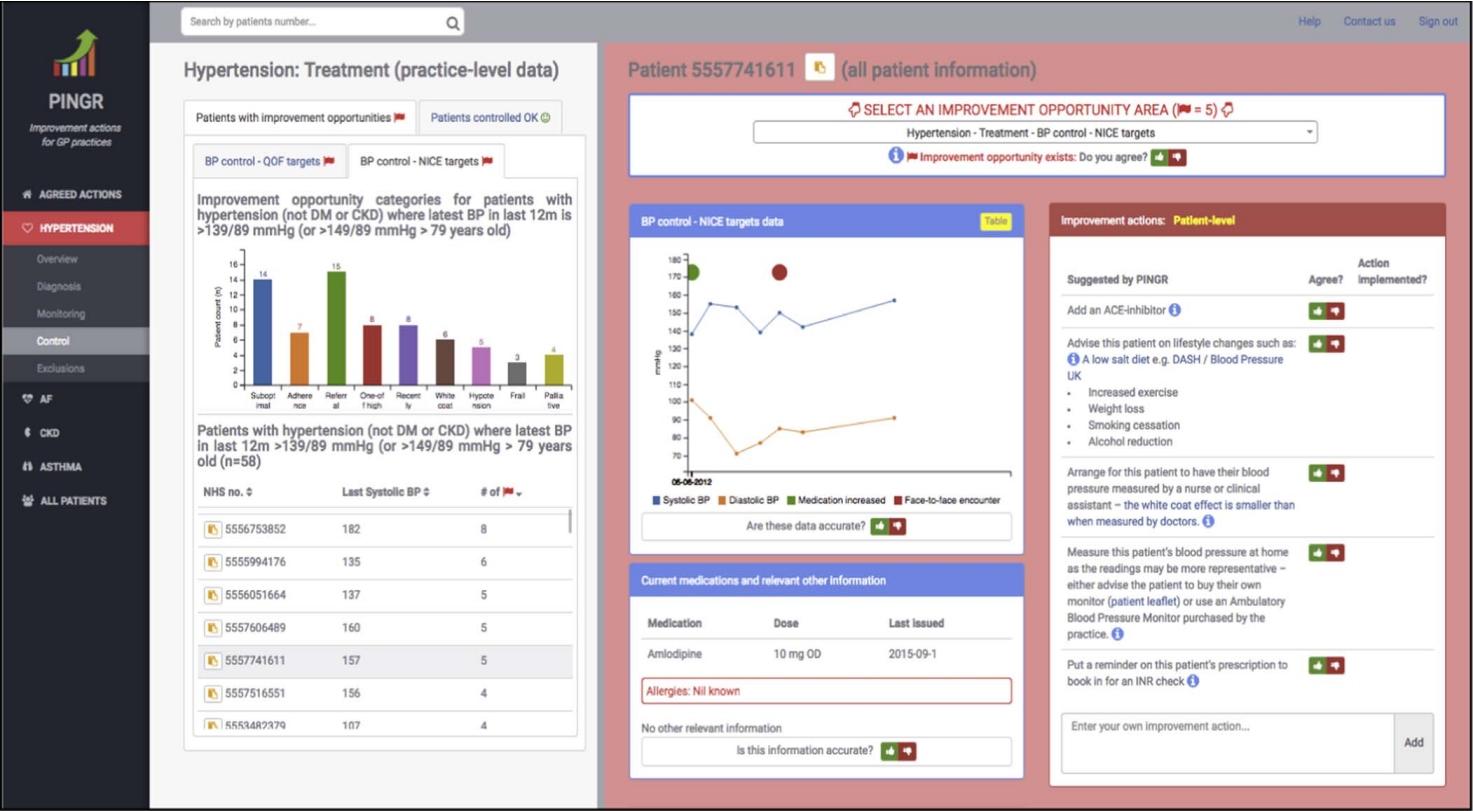
**The Performance Improvement plaN GeneratoR.** Overview level interface (top) displaying clinical performance summaries (light blue border boxes at the centre of the screen, where each box represents a care pathway: diagnosis, monitoring, control and exclusions) and organisation-level suggested actions (light red border box, right-hand side of the screen). Preview level interface (bottom) displaying the improvement opportunities bar chart, patient lists, detailed patient-level data and suggested actions. The background colour of the detailed patient-level data interface component turns red when an improvement opportunity is present. AF = atrial fibrillation; A/C = anticoagulation; BP = blood pressure; DASH = Dietary Approaches to Stop Hypertension; NICE = National Institute for Health and Care Excellence; OD = Once daily; QOF = Quality and Outcomes Framework. (For interpretation of the references to colour in this figure legend, the reader is referred to the web version of this article.)

PINGR is an e-A&F system for primary care professionals, developed by the authors (Fig. 1): a primary care physician/informatician (BB), a software engineer/informatician (RW), and a human-computer interaction expert (PB). PINGR is a web-based application that stands alone outside clinical systems. It analyses structured data extracted from electronic health records (EHRs) on a nightly basis against clinical standards and patient outcomes (e.g. from clinical guidelines).

PINGR’s present interface design was informed by a usability inspection study [3], and an emerging theoretical causal model of effective audit and feedback [15], [16]. The use of theory is recommended in the design of complex interventions in general [7], and of e-A&F tools specifically [17]. Our approach is informed by an ongoing systematic meta-synthesis of qualitative studies [15], and draws on: existing theories (such as Control Theory [18] and Feedback Intervention Theory [19]); intervention description frameworks (e.g. [20]); and organisational implementation models (e.g. [21]). The remainder of this section presents a detailed account of the design and rationale of PINGR’s four main interface components.

#### 2.1.1 Clinical performance summaries

The PINGR interface (Fig. 1) employs the overview-preview principle to display information at different levels of detail based on Shneiderman’s visual search mantra [22]. Presenting an overview of clinical performance data with details on demand was found to be an important usability feature in e-A&F systems [3]. The overview is provided as performance summaries at the level of the primary care practice/office (Fig. 1; top), where quality indicators are grouped into separate data representation modules for each clinical area. This module oriented design was employed to: enhance information processing, as is usual practice with clinical guidelines [23]; and facilitate user workflow [24]. Within each clinical area, quality indicators are further grouped into common care pathways associated with long-term care: diagnosis, monitoring and control [25], with an additional exclusions pathway to track patients excluded from the quality standards for clinical reasons (e.g. terminal illness). The purpose of the pathway groupings is to create a framework for representing a variety of clinical conditions consistently – as recommended in design best practice for EHRs [26] and clinical decision support (CDS) systems [27].

Currently, PINGR supports four clinical areas: hypertension, asthma, chronic kidney disease, and atrial fibrillation (AF). These clinical areas were chosen because they are:

1. managed mostly in primary care, making them familiar to end-users;
2. common components of multimorbidity – a major quality/safety issue in primary care [28] and core to the challenge of summarising patient information across multiple clinical areas [29], which is often not addressed by CDS systems [30];
3. often poorly cared for, resulting in serious adverse outcomes for patients and financial impacts on the health system (e.g. [31]), so address a quality improvement need;
4. associated with different quality indicators from different guidelines (e.g. process and outcome measures [25]; quality problems of overuse and underuse [32]; competing recommendations), so are suitable exemplars from which to generalise findings; and
5. covered by other commonly used existing e-A&F systems, enabling users to make comparisons with PINGR about its relative (dis)advantages.

As prescribed by cognitive fit theory [33], quality indicators are displayed as separate modules for tables and line graphs to support both symbolic and spatial information processing respectively. Both involve perceptual and analytical processes that are fundamental to the interpretation and understanding of quantitative health information [34]. In addition, providing users the option to select between tables and line charts is in accordance with mainstream usability heuristics (e.g. [35]), and effective audit and feedback data presentation theories [4], [36]. Tables are presented by default, which users can switch to display time trends as line graphs. The rationale for displaying tables first rather than line graphs was that the addition of rows to tables would more easily facilitate the expansion of PINGR to include further quality indicators, whilst at the same time facilitating users to easily interpret their current clinical performance [33]. Although many e-A&F systems explicitly compare user’s quality indicator scores to targets/goals [3], evidence for their use in improving feedback effectiveness is mixed [2]. Therefore in accordance with actionable feedback design [37], PINGR non-judgmentally presents clinical performance data to users which they can compare with their past performance on line charts, and their internal beliefs regarding care quality [19].

Using a menu on the left side of the screen, users can display performance summaries for a specific clinical module at the overview level (e.g. AF; Fig. 1; top), or proceed directly to the preview level to view more detail by selecting a care pathway (i.e. diagnosis, control, treatment or exclusion; Fig. 1; bottom). Users can also access the preview level (Fig. 1; bottom) by selecting one of the pathway’s data representation modules in the overview interface (Fig. 1; top, blue coloured rectangular areas). At the preview level, information is presented regarding all patients who have not achieved the quality indicator and for whom improvement opportunities have been identified (Fig. 1; bottom, left side). For example, for hypertension control, these are patients whose latest blood pressure in the past 12 months is above 139/89 mmHg or above (or 149/89 mmHg if they are >79 years old) based on national quality standards [38]. An interactive bar chart shows the improvement opportunities identified for patients (Fig. 1; bottom, left side above the list). By selecting a bar within the chart, the user can generate a list of all patients in whom the improvement opportunity is relevant, for example, all patients currently prescribed suboptimal medication, or those that may have medication adherence issues. Each improvement opportunity is accompanied by an explanation as to what it refers, and its eligibility criteria, communicated via short static notes at the top of the patient list and tooltips. The use of static notes and tooltips were found to be important for users to complete the goal-action sequence of data interpretation tasks in the context of e-A&F systems [3]. A user can switch from patients who have *not* achieved the quality indicator (“patients with improvement opportunities”), to those who have (“patients OK”) using the corresponding tabs at the top of the bar chart. The user can also use a separate set of tabs to select different quality indicators relevant to the clinical pathway. For example, in hypertension control there are different blood pressure targets recommended by different organisations (e.g. [38], [39]). For each generated list of patients, users can view detailed patient-level data by selecting a specific patient identification number (Fig. 1; bottom, right-hand side). Patient lists, detailed patient-level information and suggested actions components of the PINGR interface are discussed in more detail below.

#### 2.1.2 Patient lists

As described above, patients achieving and not achieving each quality indicator are listed in the preview interface for each care pathway module (Fig. 1; bottom, left side). These lists can be ordered by patient-level clinical variables to enable users to prioritise high-risk patients, which may improve the effectiveness of e-A&F [40]. For example, patients with improvement opportunities in their hypertension care can be ordered according to their last systolic blood pressure reading. In addition, following PINGR’s usability inspection study [3] the current version includes further variables such as patients’ identification number and their number of improvement opportunities. As explained in Section 2.1.2, the lists of patients not achieving a quality indicator can be filtered by clicking the improvement opportunity bar chart (Fig. 1; bottom, left side above the list), which displays the number of patients in relevant categories (see Section 2.1.4 below for a more detailed explanation of how these categories are derived). This chart acts as an interactive visual query mechanism to list patients requiring similar improvement tasks, thus minimising user cognitive load by grouping together patients that require the same clinical action [41], [42]. Finally, an “All patients” list presents all patients within PINGR across all quality indicators combined.

#### 2.1.3 Detailed patient-level information

Detailed patient-level information can be displayed adjacent to patient lists (Fig. 1; bottom, right side). Both patient lists and patient-level information are displayed concurrently to facilitate user’s anticipated workflow of efficiently selecting new patients requiring improvement action [43]. Patients can be selected to display their information from the lists or via a search box at the top of the page. To improve system status visibility [35] as suggested from our usability inspection study, the patient-level information is separated from the patient list by a border, and when a new patient is selected a self-healing fade indicates their data is presented [3]. At the top of the patient-level information component a dropdown menu provides access to information relevant to each quality indicator. For example, selecting the blood pressure control indicator displays patient’s blood pressure measurements, whereas selecting the atrial fibrillation anticoagulation monitoring displays their International Normalised Ratio (INR) readings. As recommended in our usability inspection study [3], these data are by default presented using interactive line charts to help users assess patient readings over time (e.g. tooltips display details in the x and y axis for each data point), and are contextualised with relevant additional non-clinical details using tool-tips and vertical markers (e.g. recent medication changes or face-to-face consultations). A toggle option is available to alternatively present these data as tables [33]. Further clinical information, including the patient’s current medications and allergies, is presented below the line charts to improve interpretation of data and suggested actions for each quality indicator (Fig. 1; bottom). This design decision is also supported by research showing that additional clinical information can improve clinician decision-making [44] and user experience [27]. As data in the e-A&F system may differ from those in the EHR [43], functionality is available for users to indicate whether or not PINGR has correctly identified an improvement opportunity for a patient, and whether patient-level data is correct, using agree (thumbs up) and disagree (thumbs down) icons.

In accordance with evidence from non-clinical dashboards [41] and CDS systems [27], quality indicators listed in the dropdown menu are colour-coded and prioritised: clinical areas in which the patient violates a quality indicator are presented first in red, those they have achieved are second in green, and indicators that are not relevant to the patient but are still within PINGR are at the bottom in grey. Colour is a reliable pre-attentive property that facilitates quick identification of information without sequential searching, which can reduce short-term memory load [45], [46]. The use of colour was identified as an important element for the unobstructive completion of tasks in the cognitive walkthrough evaluation of an earlier version of PINGR [3]. The purpose of presenting data related to achieved and irrelevant indicators is to enable users to highlight if PINGR incorrectly classifies a patient (false negatives), in order to improve its algorithms [47] and support error prevention [27].

#### 2.1.4 Suggested actions

The defining feature of PINGR is that it suggests care quality improvement actions that users could take (a feature usually seen in point-of-care CDS not e-A&F systems [16]), which we call ‘decision-supported feedback’ [3], [16]. PINGR provides two types of suggested actions to users based on their specific clinical performance [48], [49]: organisation-level and patient-level. This is because: evidence suggests that both types are required for effective improvement action [50]; health professionals have difficulty and limited time to develop their own improvement actions [40]; and providing suggested actions alongside feedback is shown to improve its effectiveness [2]. Organisation-level suggested actions relate to steps that the primary care practice/office team could take collectively to improve, such as introducing a new service or changing the way they work. In the PINGR interface these are presented at the overview level, on the same page as the clinical performance summaries showing quality indicators across the four pathways (diagnosis, monitoring, control, and exclusions), and relate to suggestions for the whole clinical area (e.g. hypertension; Fig. 1; top, right side). Patient-level suggested actions relate to changes clinicians could make in caring for individual patients, such as introducing new medication, or providing lifestyle advice. They are presented alongside the detailed patient-level information component, with different suggested actions for each quality indicator accessed via the dropdown menu (Fig. 1; bottom, right side). Organisation and patient-level suggested actions are positioned to the right-hand side of the overview and preview interface respectively to match the anticipated user workflow of data interpretation and action according to both Control Theory [18], CDS design guidelines [51] and findings from our usability inspection study [3]. Furthermore, this complies with CDS design recommendations for providing relevant patient data alongside alerts [51], [52].

We have previously published an early version of our methodology for deriving patient-level suggested actions [31]. In brief, this involves translating relevant clinical guidelines into rule-based algorithms to analyse the EHR data for each patient that has not achieved the quality indicator [31]. For example, in hypertension control one suggested action concerns medication optimisation: data are analysed to derive an up-to-date medication list, which is then compared with published maximal doses [53] and clinical pathways [38]. If a patient’s current medication dose is sub-maximal, then PINGR suggests increasing the dose. Similarly, if their medication does not match the prescribed clinical pathway, then PINGR suggests starting the most appropriate medication. The algorithms also take into account contextual information about patients [51], such as relevant comorbidities and allergies, by not suggesting medications for which they have a contraindication (e.g. PINGR would not suggest a beta blocker for a patient with asthma). These patient-level actions form the basis of the categories in the improvement opportunity bar chart (Fig. 1; bottom). In this version of PINGR, organisation-level actions were derived from quality improvement actions in the wider literature and clinical guidelines (e.g. [54]).

To improve help and documentation [3], [55], information buttons provide explanations for how suggested actions were generated. Hyperlinks to case reports of how other organisations had achieved change and other useful clinical tools (e.g. patient information leaflets) are also provided. These were designed to make the suggestions more actionable by providing further information on demand [51], and drawing on Social Proof Theory [56]. Users can agree or disagree with PINGR’s suggested actions by clicking thumbs up or thumbs down icons respectively. When the thumbs up icon is clicked, the action is saved to a separate (“agreed actions”) page where it can be viewed, downloaded to share with colleagues, and marked as “implemented”. When the thumbs down icon is clicked users are asked why they disagreed with the action, using optional fixed and free-text responses [26], [51]. As guided by CDS literature this is intended to communicate that the recommendations are advisory, in order to improve system acceptability and potentially system effectiveness [51], [57], in addition to collecting information on how PINGR’s algorithms could be improved [47]. Users can also add their own actions in addition to the ones suggested by PINGR, which is intended to increase user control and freedom [35], and build a user-sourced bank of suggestions.

Additional functionality suggested by PINGR’s usability inspection study [3] included: use of consistent and concise statements to avoid misinterpretation (all suggested action statements were written by BB and pilot-tested with two additional clinicians); improved visibility of system status (e.g. by showing clearly when a specific action was agreed by turning green, disagreed by turning red and disappearing, or implemented by using strikethrough text); prevention of errors by disabling further editing of an action once marked implemented; supporting user control over actions that have been agreed, disagreed or implemented (including user-generated actions) by enabling undo/redo and edit capabilities; and presentation of all suggested actions in a consistent manner, using the same typographic features and layout characteristics.

### 2.2 Participants and setting

To evaluate PINGR’s usability we recruited a sample of primary care physicians (our intended end-user group) to interact with its interface whilst performing a set of tasks. We used purposeful sampling [58] to approach physicians that would typically be expected to use PINGR in the real world through professional networks of lead author BB. A request was made either by phone, email or face-to-face to participate in a study about the evaluation of a novel web-based e-A&F system aimed at improving the quality of primary care. Physicians were eligible if they regularly used: web applications on laptop or desktop computers; EHRs; clinical decision support systems; and e-A&F systems. Eligibility was determined using a short screening questionnaire (Appendix A), which was sent via email along with an information sheet about the study. A good level of familiarity was determined in terms of number of years in practice (at least three years as primary care physicians), frequency of computer and internet use (at least five to 10 h per week), and use of specialised health care software at work (at least half the days).

Participant recruitment was conducted concurrently with data collection and analysis. Our target sample size was between five to ten participants to balance costs and maximise usability issue discovery [59], [60]. We stopped recruitment when thematic saturation was reached, which we defined as no new usability issues arising after two further participants [61]. Applying this criterion, seven physicians in total were approached and recruited to participate in the study (the sample’s characteristics are presented in Section 3.1).

Testing took place at the usability laboratory of the School of Computer Science of the University of Manchester, and was conducted by author BB. At the beginning of each test participants were briefed about the study objectives and usability test protocol, then asked to sign a consent form. During briefing participants were given a short standardised description of PINGR’s functionality, though no demonstration or training was provided. Participants then completed two background questionnaires measuring their level of graphical literacy [62] and numeracy skills [63] as both characteristics could influence participants’ interaction with PINGR and therefore help understand any differences in user interaction. PINGR was accessed via Google Chrome browser on a desktop Windows computer with a 17-in. screen. For information privacy reasons the version of PINGR used in the tests used only simulated patient data. Participants were offered re-imbursement for their time (£50) plus associated travel costs. The study was approved by the UK National Research Ethics Service (Harrow; reference 15/LO/1394) and Greater Manchester Clinical Research Network (reference 187283).

### 2.3 Tasks and task administration

Participants completed 7 tasks using PINGR (Appendix B) in a within-subjects design. As shown in Table 1, tasks were designed to assess participants’ interaction with all interface components of PINGR using realistic actions users would perform with an e-A&F system based on existing literature [15]. Specifically, tasks reflected both behavioural and cognitive aspects of user interaction with the interface. To understand the effect of interface design on participants’ cognition, Tasks 1, 2, 3 and 5 required multiple perceptual and cognitive sub-tasks including data interpretation (at both the organisational and patient-levels), and judgment of the appropriateness of PINGR’s suggested actions. Tasks 4, 6 and 7 were focused on exposing behavioural aspects of user interaction, such as locating specific information on the screen, entering data and creating and downloading user-generated actions. Tasks were presented in a randomised sequence (using the sample command in R [64]) to mitigate the effects of learning transfer, except for Tasks 6 and 7, which for logical reasons were always last. Each task was presented on-screen with contextual background information about a fictional primary care practice/office, and a patient as necessary, which participants used to inform their judgments during the tasks. To test the process of participants disagreeing with PINGR’s suggested actions and patient-level data, some were phrased to purposefully violate good clinical practice (e.g. suggesting a medication to which the patient was allergic, or presenting inaccurate patient information). To minimise participants acting unnaturally because they felt judged on their performance using the software [65], it was made clear that it was PINGR (not they) who were under evaluation.

**Table 1:** **Overview of tasks performed by participants**.

#	Description	Interface components assessed	Evaluated aspects of human cognition/behaviour
1	Interpret feedback and organisation-level actions across multiple quality indicators	Clinical performance summary Suggested actions	Identification and interpretation of relevant clinical performance summary Judgment of organisation-level suggested actions
2	Interpret feedback and patient-level actions regarding a single quality indicator	Clinical performance summary Patient lists Detailed patient-level information Suggested actions	Identification of relevant patient list Identification of appropriate patient from list Interpretation of detailed patient-level information (single disease) Judgment of patient-level suggested actions (single disease)
3	Interpret feedback and patient-level actions regarding an individual patient	Detailed patient-level information Suggested actions	Identification of relevant patient Interpretation of detailed patient-level information (multiple diseases) Judgment of patient-level suggested actions (multiple diseases)
4	Add a user-generated suggested action	Clinical performance summary Suggested actions	Identification of relevant suggested action area Data input
5	Identify the patient with the most improvement opportunities	Patient lists Detailed patient-level information	Identification of relevant patient list Identification of appropriate patient from list
6	Download saved actions	Suggested actions	Identification of saved actions download function
7	Indicate an action plan has been implemented	Suggested actions	Identification of implemented actions function

### 2.4 Data collection

We measured usability in terms of efficiency (the time taken for participants to complete each task); errors (task completion rate, and the type and number of errors made); and user satisfaction with the interface design [66]. In addition, we used utility as a fourth outcome [66] based on the number of suggested actions agreed and disagreed with while performing the tasks, and participants’ responses during interviews. Data were collected using a multi-method approach, including observation of user on-screen and visual search behaviour, post-test satisfaction questionnaires, and in-depth debriefing interviews.

#### 2.4.1 User observation

We used Tobii Pro Studio with a Tobii T60 eye tacker to record participants’ on-screen behaviour, eye movements, and time taken for completion of specific tasks. The Tobii T60 eye tracker permits a 60-Hz sampling rate, 0.5 degrees gaze point accuracy, and free head motion, which was recalibrated before each task. Author BB observed concurrently using a second monitor and took field notes, which permitted identification of interesting aspects of user interaction that were discussed during debriefing interviews.

#### 2.4.2 Post-test questionnaires

Following task completion, participants completed two usability questionnaires. We were unaware of any questionnaires specific to e-A&F systems, and therefore used the System Usability Scale (SUS) [67], and developed a questionnaire based on Shneiderman’s Object-Action Interface model (Appendix C). The SUS is a validated questionnaire that measures users’ overall satisfaction with a system’s interface [67]. It is interface agnostic and consists of 10 items with total scores ranging between zero and 100 [68]. Our Object-Action Interface questionnaire consisted of two parts aimed at evaluating specific aspects of PINGR’s user interface design: the first contained seven items regarding the ease or difficulty participants experienced undertaking actions during tasks; the second contained eight items assessing the clarity of PINGR’s interface objects (e.g. presentation of data or use of colour and terminology). Both parts used a Likert scale from 1 to 5, with 1 representing difficult or unclear, and 5 indicating easy or clear.

#### 2.4.3 In-depth debriefing interviews

Finally, participants were interviewed about their experience using PINGR after completing the questionnaires. Interviews were semi-structured (Box 1), and focused on the strengths, weaknesses, opportunities for improvement, and threats of using the software (SWOT). Questions explored concepts from Normalisation Process Theory (coherence, reflexive monitoring, cognitive participation, and collective action), which seeks to understand the work that people do, individually and collectively, surrounding a particular practice (e.g. using PINGR) rather than simply their beliefs or attitudes [69]. Other questions explored problems encountered during completion of tasks, negative responses to questions in the post-test questionnaires or other relevant additional topics that arose during interviews. As necessary, participants were replayed sections of their recorded on-screen interaction to clarify issues, and encouraged to further explore the PINGR interface. Interviews ended when both the interviewee and interviewer agreed all important topics had been covered. Interviews were audio-recorded and transcribed verbatim, and all participants were offered the option of reviewing their transcripts prior to analysis. Field notes were kept throughout the process.

Box 1 **Interview schedule.**

*Concepts from Normalisation Process Theory* [69] *addressed by each question explored in square brackets.*

Opening question: How did you find using PINGR?

Strengths.

• What are the advantages of PINGR? [Coherence]
• How useful or valuable do you think it would be in your primary care practice/office? [Cognitive participation]
• What, if anything, does it offer over existing systems you use? [Coherence]

Weaknesses.

• What are the weaknesses of PINGR? [Coherence]
• What would be the disadvantages of using it in your practice/office? [Reflexive monitoring]

Opportunities.

• How do you think you would use PINGR in your practice/office? [Collective action]
• How could it be improved in order to become a routine part of patient care processes? [Reflexive monitoring]
• How does PINGR differ from audit systems you currently use? [Coherence]

Threats.

• What are the potential threats to PINGR not being used in practice/office? [Cognitive participation/collective action]
• What problems may arise with it being used? [Cognitive participation/collective action]
• How does PINGR align with the goals of your practice/office? [Coherence]

Closing question: What have we missed that you think we should also discuss regarding PINGR?

### 2.5 Data analysis

Data analysis was concurrent with data collection. This enabled exploration of important emerging concepts in later interviews [70], and to recognise when thematic saturation had been reached [61]. Data were integrated from screen recordings, eye movements, questionnaire responses, interview transcriptions and field notes, in order to identify usability issues with PINGR in relation to its main interface components. The rationale for this approach was to: gain a more *complete* account of users’ interactions with PINGR; *enhance* and *explain* the findings of each constituent method; and *triangulate* their results to increase validity [10].

**Fig. 2:**
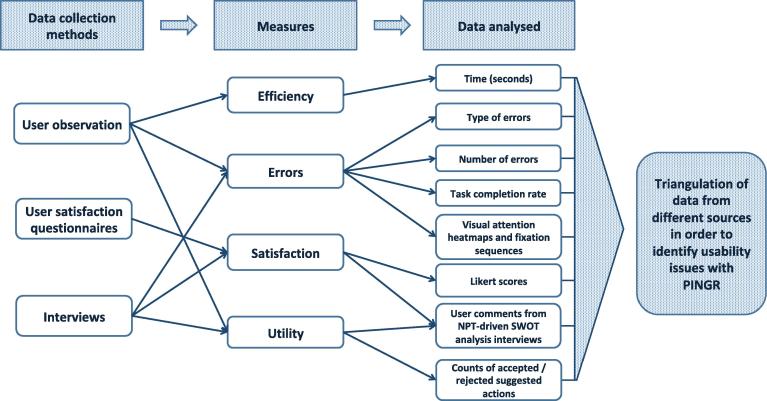
**Summary of the data collection and analysis process.** NPT = Normalisation Process theory, SWOT = Strengths, Weaknesses, Opportunities, Threats.

Fig. 2 shows a summary of the data collection and analysis process with respect to the concepts they primarily measured (i.e. efficiency, errors, satisfaction, and utility). However, often data sources were used to illuminate findings beyond these primary measures e.g. interview findings often provided insights into errors observed during user observation. To mitigate our results portraying an overly positive view of PINGR [71], our emerging analysis was critically reviewed by and agreed between our entire multidisciplinary research team (i.e. BB – primary care physician and health informatics researcher, PB – human-computer interaction expert, RW – software engineer, MS – statistician, and IB – clinical and public health informatician). This encouraged reflexivity, and increased credibility of our findings [72]. We used medians rather than means as our measure of central tendency given the small sample size and presence of outliers [73].

#### 2.5.1 User observation

Videos of participants’ interaction with PINGR (i.e. on-screen activity and visual search behaviour) were imported into NVivo 11 (QSR International) for analysis with respect to efficiency, errors, and utility [66]. Efficiency was calculated as the time taken for participants to complete each task. Errors were defined as deviations of actual from expected user behaviour. A thematic content analysis [74] determined the number and type of errors by categorising them according to CDS system usability design heuristics [27], and the interface component to which they related. We calculated the total number of errors performed by users during each task, in addition to each task’s completion success rate. Utility was calculated as the number of suggested actions users agreed and disagreed with. Eye movement data in error-prone tasks were analysed in Tobii Pro Studio to understand the attention paid to areas of interest (AoIs). We defined six AoIs according to the key interface components of e-A&F systems; two at the overview level (Fig. 1; top; clinical performance summaries, and organisation-level suggested actions), and four at the preview level (Fig. 1; bottom; improvement opportunities bar chart, patient lists, detailed patient-level information, and patient-level suggested actions). We used heatmaps to visualise the number and duration of fixations on the interface, and collapsed fixation sequences to understand how participants transitioned between AoIs. Transition matrices presented the probability of participants transitioning a fixation from one AoI to another [75]. Because the tasks used in this study included both reading and visual searching a fixation was defined as a stable gaze lasting at least 100 ms [76]. Any fixation lasting less than 100 ms was recorded as a saccade, i.e. a rapid eye movement between two fixations where no new information is processed by the participant [76]. When interpreted in conjunction with the efficiency, errors and utility data, heatmaps and transition matrices provided insights into participants’ workflow pattern and the appropriateness of how PINGR’s interface components were organised.

#### 2.5.2 Post-test questionnaires

Data from post-test questionnaires were analysed in R [64]. Statistics included median, range, and upper and lower quartiles.

#### 2.5.3 In-depth debriefing interviews

Interview transcripts and field notes kept during the interviews were imported into NVivo 11 (QSR International) for thematic content analysis [74]. Data items were coded line-by-line by author BB to create a set of themes that explained user perceptions of the PINGR tool. These themes were organised into a framework based on the SWOT analysis at the highest level, with lower level codes relating to PINGR’s four interface components, NPT constructs, and usability heuristics [27]. The process was iterative in that each data item was reviewed multiple times to refine themes and codes. Findings were discussed with and critically reviewed by PB [72]; any disagreements were resolved through discussion.

## 3 Results

### 3.1 Participants

Tests took place during September and October 2015, and took between 1.5 and 2 h per participant. Each participant (2 female, 5 male; age range 25–64 years) had between 6 and 33 years’ experience as a medical doctor, 3 and 28 years’ experience as a primary care physician, and 5 and 25 years’ experience undertaking audit and feedback. All participants used EHRs and CDS systems daily at work, felt at least 70% confident in their numeracy skills (e.g. using fractions, percentages, and graphs) [63], and scored at least 85% on the graphical literacy test [62]. All participants used e-A&F systems, though less often than EHRs and CDS systems: one participant used them “nearly every day”, with the rest using them “half the days” (n = 3) and “less than half the days” (n = 3). None of the participants had used PINGR previously, or had visual impairments that would affect the quality of eye movement recordings.

### 3.2 Efficiency

**Fig. 3:**
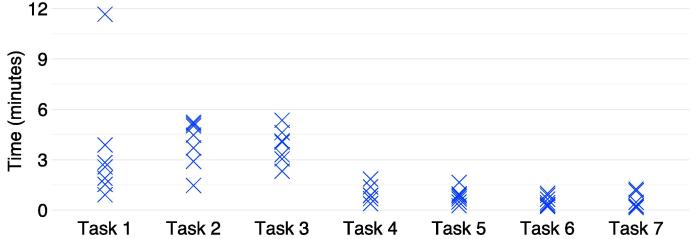
**Time taken to complete each task by participants.** Each cross represents one participant.

Fig. 3 shows the distribution of time spent by participants on each task. Tasks 1–3 (1. interpret organisation-level feedback/actions; 2. interpret patient-level feedback/actions – single indicator; and 3. interpret patient-level feedback/actions – multiple indicators) were the most time consuming, which could be because they were the most complex. Although Task 5 (identify high-priority patient) also required multiple perceptual and cognitive sub-tasks, these were limited to a single data variable (number of improvement opportunities). Conversely, Tasks 1–3 required interpretation and judgment of data relating to either organisation-level performance or patient-level clinical variables, both of which are multi-dimensional, in addition to their corresponding suggested actions. Task 2 had the highest median completion time overall (4.5 min), though one participant during Task 1 spent the longest time across all tasks (11.7 min).

### 3.3 Errors

**Fig. 4:**
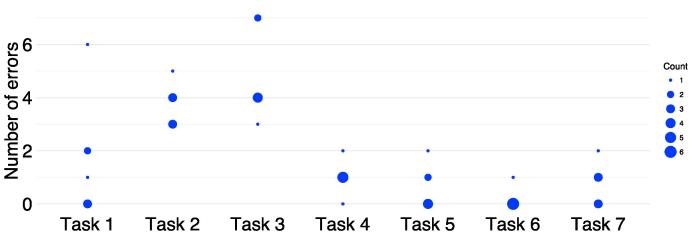
**Number of errors made during each task by participants.** The size of the dot represents the number of participants who committed that number of errors.

Participants committed a median of 10 errors (range 8–21) associated with PINGR’s key interface components during all tasks, and completed a median of 5 out of 7 tasks (range 4–7). Fig. 4 shows the error frequency distribution for participants during tasks: it mirrors the task duration distribution (Fig. 3) in as much as Tasks 1–3 (1. *interpret organisation-level feedback/actions;* 2. *interpret patient-level feedback/actions – single indicator;* and 3. *interpret patient-level feedback/actions – multiple indicators*) were associated with the most errors. These tasks also had the lowest completion rates: Task 3 was lowest (1 participant completed), followed by Task 2 (2 participants), and Task 1 (5 participants); all participants completed tasks 4–7.

**Fig. 5:**
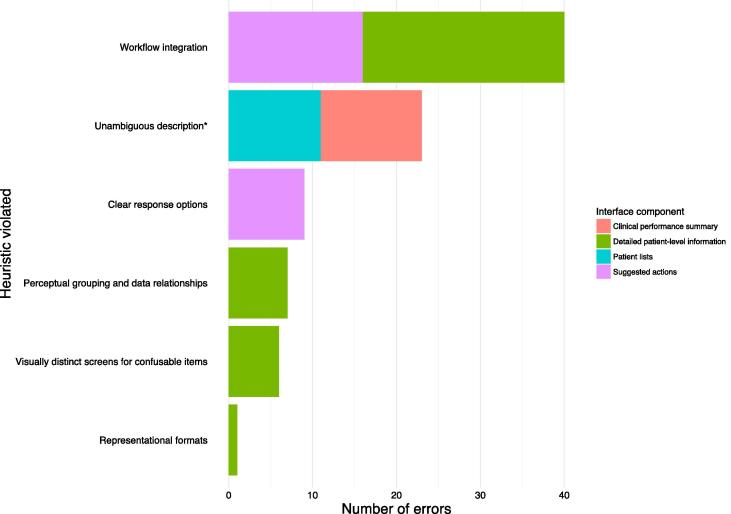
**Number of errors according to usability heuristics****[27]****and interface component.** * “Unambiguous description” category was derived by combining “unambiguous units” and “concise and unambiguous language” [27].

Fig. 5 shows the usability heuristic each error violated, and which interface component it concerned. Six (16%) out of a possible 38 heuristic categories were violated [27]. The most frequently violated was workflow integration (n = 40), all of whose errors concerned the detailed patient-level information (n = 24) and suggested actions (n = 16) interface components. With respect to detailed patient-level information, participants (n = 4) did not interact with thumb icons to indicate whether PINGR had correctly identified an improvement opportunity, or whether the information presented was accurate. They explained during interviews they would not have time to check these during their busy clinical schedules. With respect to suggested actions, participants (n = 5) did not indicate agreement with those they added themselves: they felt this should be automatic to save time – although they may disagree with an action to demonstrate it had been considered, the default would be agreement.

Other important errors related to: unclear response options when undoing a prior action (dis)agreement or marking an action implemented (n = 9); problems with perceptual grouping and data relationships caused by the dropdown menu when accessing detailed patient-level information (n = 8); visually indistinct screens for confusable items when a new patient was displayed (n = 6); and difficulties interpreting line charts as representational formats for one-off or low readings of physiological parameters (n = 1).

**Fig. 6:**
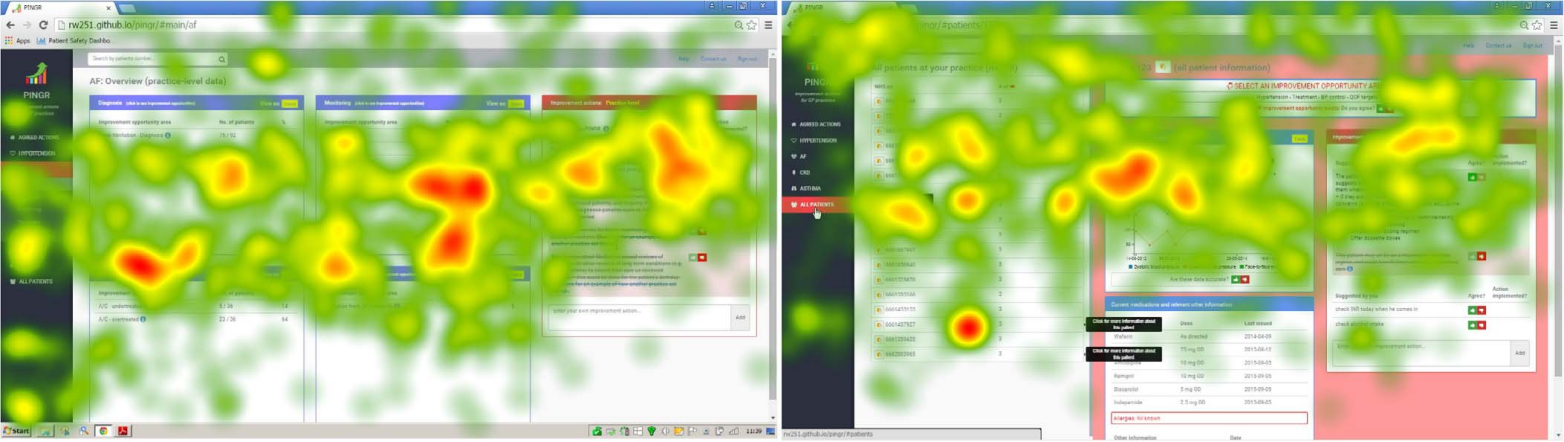
**Visual attention heatmap for Task 2 (interpret patient-level feedback/actions – single indicator).** Overview page is left; preview page is right. Red represents more fixations whereas orange, yellow and green progressively less. In this task users were asked to: select a specific quality indicator on the overview page to take them to the preview page; then at the preview page select a patient from the list, and interpret the patient data and suggested actions. Consequently, more fixations would be expected on the preview page (right) because the majority of the task requires activity here, however, there are more fixations on the overview page (left). (For interpretation of the references to colour in this figure legend, the reader is referred to the web version of this article.)

**Fig. 7:**
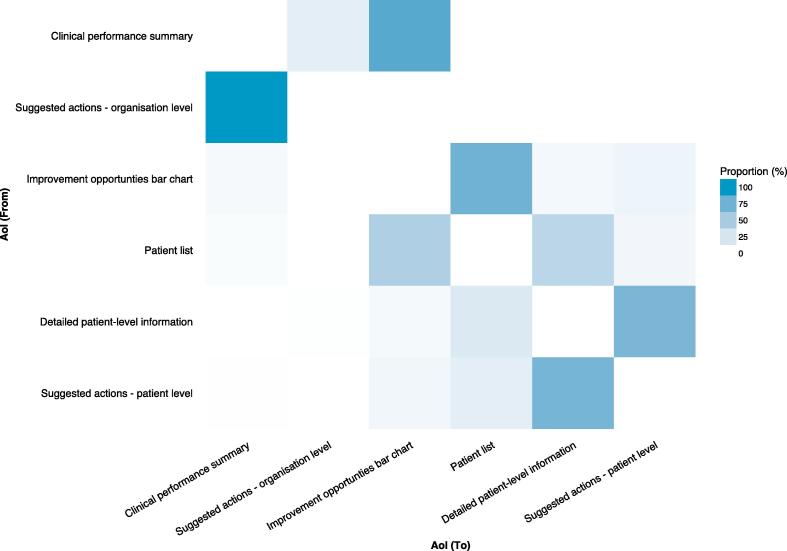
**Transition matrix of eye movement sequences between areas of interest during Task 2 (interpret patient-level feedback/actions – single indicator).** Number of visual transitions from one AoI to another as a proportion of transitions to all other AoIs. In this task users were asked to: select a specific quality indicator (clinical performance summary); select a patient from the list (patient list); interpret the patient data (detailed patient-level information) and suggested actions (suggested actions – patient-level). Consequently, high proportions of transitions would be expected between these AoIs to mirror the task completion sequence. However, there are also high proportions of transitions between AoIs not in the sequence e.g. from ‘Clinical performance summary’ to ‘Suggested actions – organisational level’, and vice versa. The axis labels are ordered based on how they would be encountered during the task. AoI = Area of Interest.

Eye movement data analysis focused on Tasks 1–3 (1. interpret organisation-level feedback/actions; 2. interpret patient-level feedback/actions – single indicator; and 3. interpret patient-level feedback/actions – multiple indicators) given they were the most time-consuming and error-prone. An example is illustrated in the heatmap for Task 2 (Fig. 6), which is similar to that observed for Task 3. Both tasks required identification of appropriate patient-level information and suggested actions at the preview page, which should result in greater visual activity at those interface components. However, the heatmaps demonstrated the opposite: a greater number of fixations on the overview page (Fig. 6). In addition, although the transition matrix of eye movement sequences for Task 2 (Fig. 7) showed high probabilities of transitions between AoIs compatible with optimal workflows for task completion, it also demonstrated unexpected transitions – particularly at the overview page. When considered together, Fig. 6, Fig. 7 suggest that although integration of AoIs at the preview level (Fig. 1; bottom) largely supported effective user interaction, the overview level (Fig. 1; top) unnecessarily increased user’s cognitive load. Typical errors at the overview page relevant to Tasks 2 and 3 included participants selecting the wrong quality indicator, which led to the wrong patient list, and ultimately the wrong patient. These violated the unambiguous description heuristic and accounted for errors at the clinical performance summary (n = 12) and patient list interface components (n = 11). Participants (n = 5) explained such errors arose because PINGR used separate modules and pathways to organise quality indicators, making it difficult to prioritise on which one to focus. Although users could view all quality indicators within the same clinical area concurrently, they could not view indicators across different clinical areas. Therefore, judging which one required the most urgent attention required accessing each module individually and comparing performance across different pathways. These problems were exacerbated because scores were not explicitly compared to desirable levels of performance (targets/goals), so making value judgments required further information processing. Participants suggested comparisons with other primary care practices/offices would be most helpful in interpreting their performance (peer benchmarking).

### 3.4 Satisfaction

**Fig. 8:**
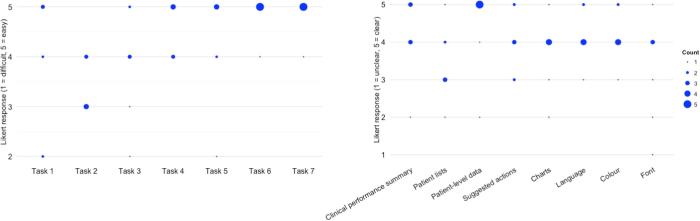
**Participant responses to the Object-Action Interface questionnaire.** Actions are left; Objects are right. The size of the dot represents the number of participants who provided that response.

The median SUS score was 73 (range 58–88), indicating a passable level of satisfaction with PINGR’s usability [68]. This was supported by interviews, where all participants (n = 7) volunteered that PINGR was easy to use. Nevertheless, some felt a tutorial module would be helpful, particularly to highlight PINGR’s novel features such as its suggested actions. Despite the number of errors observed, Fig. 8 shows that overall participants felt tasks were easy to complete: the lowest median Likert difficulty rating for any task was 3, and no participant gave the lowest score of 1. Task 2 (*interpret patient-level feedback/actions – single indicator*) was reported the most difficult (median Likert rating = 3, range = 3–4), with Tasks 1 and 3 (1. *interpret organisation-level feedback/actions;* and 3. *interpret patient-level feedback/actions – multiple indicators*) joint second (median Likert ratings = 4, and ranges = 2–5). This mirrors findings regarding efficiency and errors described above (3.2 and 3.3, respectively). Participants who committed the most errors in Tasks 1, 3 and 5 (*identify high-priority patient*) rated them as most difficult.

Fig. 8 also shows that participants felt in general that PINGR’s interface was clear, with only one participant giving the lowest Likert rating of 1 because they felt the font was too small. The patient lists interface component was considered least clear (median Likert rating = 3, range = 2–5): participants (n = 3) wanted further clinical variables to prioritise patients despite the additions made following PINGR’s previous evaluation described in Section 2.1.3 above [3]. Suggestions included: patients’ age, as younger patients would likely gain most benefit from clinical actions; risk of a relevant outcome (e.g. cardiovascular disease event in the blood pressure control quality indicators); and whether patients had violated a particularly high risk quality indicator (e.g. inappropriately untreated AF). Three participants stated the number of patients in each list was overwhelming, and that in practice they would likely not have the time or resources to review each one. They would deal with this by focusing on the most high priority patients, and by sharing lists with other staff (e.g. printing lists for a nurse to examine on their behalf). A further problem related to the use of red flag icons to denote the number of improvement opportunities. This was confusing because in clinical medicine red flags usually refer to important clinical signs and symptoms. As an alternative they suggested using a different colour or icon (e.g. a lightbulb, star or lightening flash).

Despite being associated with the most errors during tasks, the detailed patient-level interface component was rated most clear by participants (median Likert rating = 5, range 2–5), with all but one participant giving the highest score of 5. During interviews, participants (n = 3) made positive comments about the benefits of visualising patients’ physiological data as line charts as it helped interpret its clinical significance, and prioritisation of patients.

Both the clinical performance summaries and suggested actions interface components received a median Likert rating of 4. With respect to clinical performance summaries, participants stated framing them positively rather than negatively was preferable because it rewarded clinicians and encouraged further action (e.g. showing the number of correctly treated AF patients, rather than the number incorrectly treated). They also suggested presenting the historic clinical performance trend data alongside the current performance as default to aid interpretation and quality indicator prioritisation, as it was unclear how to access it via the toggle buttons. Similar suggestions were also made regarding the improvement opportunities bar chart.

With regards to suggested actions, all participants stated they were acceptable because they were presented as suggestions (rather than diktats) and could be disagreed with. The majority (n = 4) were concerned they would not have the time or resources to evaluate and implement every suggested action, and would only be able focus on the most important. However, they felt this would be difficult because the modular format of the clinical performance summaries prevented viewing organisation-level actions across multiple quality indicators concurrently, as did the dropdown menu in the detailed patient-level information component for patient-level suggested actions. Participants stated they only wanted to be presented with the top three or four most important improvement actions they could implement, and that ideally PINGR should make this judgment for them. They suggested actions’ importance could be ranked according to: clinical safety (e.g. high-risk drug-drug interactions requiring urgent attention); potential effectiveness (e.g. predicted impacts on patient outcomes); financial value (e.g. how much money the organisation could save from unnecessary laboratory tests); and quick wins (i.e. the perceived ratio of implementation effort to potential benefit – either clinical or financial). Almost all participants (n = 5) stated positioning suggested actions on the right of the page was satisfactory as it mirrored their workflow of data interpretation then action plan formulation. However, one participant failed to complete a task because they did not visualise them. In the same vein, participants recommended moving the “agreed actions” page (where their agreed actions were saved) to the end of the navigation menu to fit with to fit their workflow of reviewing action plans last. Other recommendations for improving suggested actions included: using less prose; providing detail on demand only; and including more specific reasoning as to why each action was suggested.

### 3.5 Utility

During tasks, each participant viewed a median of 12 suggested actions (range 5–13), of which they agreed with a median of 7 (range 4–8), disagreed with a median of 3 (range 0–6), and did not respond to a median of 0 (range 0–8). Reasons for disagreements with organisation-level suggested actions were: prior implementation in the participant’s organisation (n = 4); a lack of implementation resources (n = 1); or a perceived inefficient use of resources (n = 1). Reasons for disagreements with patient-level suggested actions related to: disagreement with a clinical guidelines’ recommendation (n = 3); absence of local services to carry out the suggestion (n = 2); lack of information regarding patients’ medication adherence (n = 1); and desire to clinically assess the patient (n = 1). Two participants did not respond to suggested actions because they wanted to defer a decision following either discussion with colleagues (regarding organisation-level actions) or individual patients (for patient-level actions), or perform other actions first. They suggested the ability to manually order and prioritise actions in the “agreed actions” page, with functionality to assign actions to other staff members with deadlines and reminders to track progress, may help with this issue and integrate PINGR with their workflows. Worryingly, two participants agreed with patient-level actions for which the patient had contraindications (i.e. patient refusal of a home blood pressure monitoring service, and prescription of a medication to which there was an allergy); on further questioning, both did not expect the system to suggest actions to which there were documented reasons against. Of their own accord, three participants added 10 actions to PINGR they had formulated themselves. These covered organisation-level actions (n = 2), such as services or quality improvement ideas not currently suggested by PINGR, and patient-level actions (n = 8) relating to lifestyle advice and medication safety issues. A summary of findings relating to PINGR’s utility from the NPT-driven SWOT analysis interviews is presented in Box 2.

Box 2 **Summary of NPT-driven SWOT analysis interviews related to PINGR’s utility.**

**Strengths**

*Clinical performance summaries*

• Covers multiple clinical areas and quality indicators relevant to primary care.
• Includes quality indicators relating to undiagnosed patients, over-treated patients (e.g. over-anticoagulation in AF), and patients who may benefit from exclusion (e.g. palliative care patients), thus addressing issues of over-medicalisation often ignored by e-A&F systems.
• Improvement opportunity chart provides unique insights into reasons for poor performance, guides improvement action, and saves time by filtering patient lists to those requiring the similar actions.

*Patient lists*

• Lists patients requiring action to facilitate quality improvement.

*Suggested actions*

• Shifts focus from data interpretation to improvement action not seen in other e-A&F systems.
• Saves time by negating the need for users to formulate their own action plans.
• Functionality to save, download and mark actions as implemented helps manage workflow, enables communication with other staff, and can be used as evidence for annual appraisals.
• Links to case reports (organisation-level) and patient information leaflets (patient-level) aids implementation.
• User-added actions share best practice between organisations.

*Detailed patient-level information*

• Ability to drill-down from population-level to patient-level data via interactive links is intuitive and user-friendly.
• Provides non-clinical data (e.g. patient contacts with primary care practice/office) alongside clinical data (e.g. historic blood pressure measurements), which contextualises how to implement improvement action (e.g. how to contact a patient to measure their blood pressure if necessary).

**Weaknesses**

*Clinical performance summaries*

• Inclusion of quality indicators with differing guidance is confusing (e.g. different blood pressure targets [38], [39]).
• Unclear how to use improvement opportunity chart to filter patient list.
• Improvement opportunity chart sometimes contained too many categories and unclear explanations to be efficiently interpreted.

**Opportunities**

*Clinical performance summaries*

• Addition of quality indicators in areas important to primary care (e.g. chronic obstructive pulmonary disease, diabetes, and general health checks).
• Tailoring of quality indicators displayed based on user-preference.

*Detailed patient-level information*

• Inclusion of: demographics; medication adherence or prescription frequency; historical medication prescriptions with dates and reasons for cessation; relevant improvement opportunity categories; and additional physiological parameters and comorbidities.

*Suggested actions*

• Integration with existing information systems, including: ability to open patients’ EHRs and vice versa; write-in functionality to EHRs; direct communication with patients via text messages/letters/emails; and medication prescribing.
• Ability to view other users’ agreed actions within their organisation to aid action planning and prevent work duplication.
• Inclusion of patient decision aids where appropriate (e.g. regarding recommended treatments).
• Alignment with local clinical pathways.
• Link organisation-level suggested actions to specific reasons for suboptimal performance.
• Present those not previously considered by users.
• Patient-level actions may be valuable for nurses conducting chronic disease clinics.

**Threats**

*Detailed patient-level information*

• Should not aim to be comprehensive because users:
  o only wish to view data relevant to quality indicators;
  o may believe they are viewing the full EHR, which may lead to safety issues (e.g. if all currently prescribed medications or laboratory tests are not displayed).

*Suggested actions*

• Some users may formulate their own actions and ignore them.

*AF = Atrial fibrillation; e-A&F = electronic audit and feedback; EHR = Electronic health record.*

## 4 Discussion

This study identified usability issues with a novel actionable e-A&F system for primary care, regarding efficiency, errors, satisfaction, and utility. The main strength was to use a multi-method approach to evaluate all four e-A&F interface components (clinical performance summaries, patient lists, detailed patient-level data, and suggested actions) to *enhance*, *explain*, *triangulate* and increase *completeness* of findings [10]. The main limitation was that the team who developed the system also performed the evaluation.

In the following discussion we combine our findings with wider usability literature in order to refine a previously published set of preliminary interface design recommendations for e-A&F systems [3] (Box 3). We also discuss the implications of these findings for patient safety by drawing on emerging results from an ongoing systematic meta-synthesis of qualitative research studies of A&F performed by our research group [15]. Finally, we discuss the limitations of our research methodology in more detail.

Box 3 **Summary of interface design recommendations for electronic audit and feedback systems, and questions for further research, refined from[3].**

*Clinical performance summaries, should:*

• Cover multiple clinical topics relevant to users.
• Address over-treatment, missed diagnoses, and situations where it may be inappropriate to treat patients (e.g. when receiving palliative care).
• Allow users to select which quality indicators to display.
• Be framed positively where appropriate to emphasise achievement (e.g. patients achieving a quality standard, rather than those not achieving).
• Be presented across all clinical topics concurrently in one display screen.
• Use line graphs to demonstrate trends over time with tooltips to interpret historic performance data.
• Compare users’ scores to desirable levels of performance (targets/goals).
• Automatically prioritise quality indicators (e.g. through the use of colour or ordering).
• Undertake further data analysis and visualisation related to improvement action.
• Explain clearly to what performance data specifically refer.

*Patient lists, should:*

• Present sufficient information to efficiently prioritise patients (e.g. age, physiological measures, number of quality indicators violated).
• Allow users to control what information is used to prioritise patients.
• Clearly allow users to order and filter patients.
• Display a limited number of high-priority patients (e.g. 10), with more on-demand.
• Use appropriate icons to communicate patient variables.
• Explain clearly to what they refer.

*Detailed patient-level information, should:*

• Be accessible via interactive links to drill-down from population-level data.
• Be comprehensive enough to provide users with sufficient information to interpret suggested actions, and formulate their own.
• As a minimum include demographics, diagnoses, physiological measures, and prescribed medications.
• Include both clinical and non-clinical data (e.g. contacts with the primary care practice/office).
• Only include information directly relevant to taking improvement action.
• Use line charts to display physiological data with tooltips to interpret historic performance data.
• Provide users the option to display data as tables.
• Be displayed on a single page.
• Be completely separate from other interface components.
• Provide a warning the system is not attempting to replicate a patient’s health record.
• Enable users to highlight inconsistencies with health record data.

*Suggested actions, should:*

• Address both the individual patient and organisation.
• Be derived from acceptable sources (e.g. clinical guidelines or the wider quality improvement literature).
• Align with local clinical pathways.
• Address specific reasons for users’ poor performance based on detailed analysis of clinical performance data.
• Adapt to contextual features of organisations (e.g. whether or not actions have already been implemented) and individual patients (e.g. potential contraindications).
• Strive to present ideas users have not previously considered.
• Be written concisely (e.g. using bullet points).
• Provide details on-demand regarding why they were suggested, how they have been implemented in other organisations (case reports), and patient-facing information (e.g. information leaflets).
• Address the first step a user may take during implementation.
• Be located in a separate interface component aligned with user workflow (e.g. on the same screen as clinical performance summaries, or detailed patient-level information).
• Display only the most important three or four options concurrently, though provide the option to view more if desired.
• Be prioritised according to patient safety, potential effectiveness, financial value, or ‘quick wins’, which should be accommodated through user settings where possible.
• Have their prioritisation communicated through the order in which they are displayed or their colour.
• Be advisory, allowing users to indicate disagreements (using both fixed and free-text responses).
• Use data from user disagreements to improve their algorithms.
• Allow users to add their own actions, which should be saved automatically, and used to optimise the system’s own suggestions.
• Allow users to clearly save, mark them implemented, and view those of other users within their organisation.
• Provide functionality to view, undo and edit previous disagreement reasons.
• Allow users to order and manually prioritise saved actions, set deadlines and reminders, assign them to users, and export for wider sharing.
• Have clear response options, and ideally automatically detect when an action has been implemented.
• Provide functionality to easily action the recommendation, which may be facilitated through integration with existing health information systems (e.g. write-in functionality to health records or direct patient communication via text message).

*Questions for further research:*

• How much interface adaptation should be user-controlled or automated?
• What methods can optimise automated interface adaptation?
• What methods are most appropriate to adapt suggested actions to contextual features of organisations and patients?
• Which are the most effective types of targets/goals to use in clinical performance summaries?
• What additional methods for deriving suggested actions are possible, acceptable, efficient, and effective?
• What are the most appropriate criteria and methods to prioritise clinical performance summaries, patient lists, and suggested actions?
• What are the optimal ways to communicate and display this prioritisation?
• What is the optimal position of suggested actions within the user interface?
• How do findings from this study translate to more naturalistic settings outside the laboratory?

### 4.1 Refined interface design recommendations for e-A&F systems

#### 4.1.1 Clinical performance summaries

Clinical performance summaries should cover multiple clinical topics relevant to users. Where possible they should address issues of over-treatment, missed diagnoses, and inappropriate treatment (e.g. in patients receiving palliative care). To align with users’ workflows they should offer functionality to include quality indicators addressed by existing quality programmes (e.g. [38], [39] in PINGR’s case), and for users to select those in which they are most interested. Where appropriate, clinical performance should be framed positively rather than negatively (e.g. by presenting the number of patients attaining a quality standard rather than the number not attaining). This is supported by studies of presenting quantitative information to clinicians [77], [78], and may leverage principles of positive psychology by making users feel recognised for their performance efforts, creating a positive loop of further action and interaction with the system [79].

Quality indicator results from all clinical domains should be presented concurrently in one display screen. This enhances information processing by making data comparison efficient, and is supported by studies of health-related dashboards [80], [81]. Data prioritisation can be further helped through the presentation of clinical performance trends over time, and by explicitly comparing users’ scores with desirable levels of performance (targets/goals). Both elements should be presented simultaneously to increase the likelihood of visualisation; the use of space-saving tools such as sparklines [82] may help. Although users may prefer peer performance data as the target/goal by which to judge their performance (e.g. average peer performance), other options for choosing targets/goals exist (e.g. set by experts, or based on past performance). Systems may further reduce cognitive load by automatically prioritising quality indicators on behalf of users and communicating this via colour (e.g. RAG rating) or the order in which they are displayed [41]. Criteria for prioritisation may include current levels of performance or predicted numbers of patient adverse outcomes [47], [51].

Like PINGR’s improvement opportunity charts, e-A&F systems should routinely evolve their approaches to the analysis and visualisation of clinical performance. This is supported by findings from evaluations of other e-A&F systems [80], [83], and may include patients not achieving quality standards grouped according to similar actions (as in PINGR), or other patient/organisation variables. Where possible, these visualisations should highlight relevant patients for action via the patient list interface component. To facilitate cognitive processing, analyses should be displayed concurrently with overall quality indicator results, and provide a limited number of findings (e.g. by focusing on the largest groups of patients). They should include clear explanations of their methods and results, with instructions regarding how they may help action planning. Such detailed instructions have been found necessary in similar population-level data exploration tools [84].

#### 4.1.2 Patient lists

Patient lists should present sufficient information to enable users to efficiently prioritise patients for action or review, which may include: age; physiological measures relevant to the quality indicator; number of relevant improvement opportunities; whether a particularly high-risk quality indicator is violated (e.g. untreated high-risk AF); and where relevant, their predicted risk of an adverse outcome (e.g. cardiovascular disease event). Given the many variables that could be included in patient lists, and variation in user preference demonstrated in our study, it may be appropriate for users to maintain freedom and control by customising which are displayed [35]. The ability to order and filter patient lists is essential for prioritisation, and the availability of this function should be made clear. User control over patient list variables and ordering is supported by wider EHR design guidelines [85]. To avoid the volume of patients in lists overwhelming users, e-A&F systems may display a manageable number at any one time (e.g. 10) starting with the highest priority, and further displayed on demand. This conflicts with EHR design guidelines that state all patients in a list must be visible on one page to ensure they are not missed [85], though is acceptable because e-A&F systems are not intended for direct patient care (unlike EHRs) [2]. Icons used in patient lists should be appropriate to the clinical context in which they are used. For example, radiological systems should avoid using red dot icons, which are also used to highlight abnormal findings on a radiological image [86]. This agrees with more general icon usability guidelines regarding [87], though may only be recognised as problematic through user testing.

#### 4.1.3 Detailed patient-level information

Detailed patient-level information should be accessible via interactive links to drill-down from population-level aggregated data [22]. It should be comprehensive enough to provide users with sufficient information to: interpret suggested actions; formulate their own; and avoid inefficiencies of accessing other information sources (e.g. EHRs). Similar recommendations have been made with primary care population-level data exploration tools [88]. As a minimum, patient-level information should include: demographics; diagnoses; physiological measures; and current and past medications (including data on adherence and reasons for stopping medications). Both clinical (e.g. physiological measurements, medication prescriptions) and non-clinical (e.g. contacts with the primary care practice/office) data should be displayed to illustrate how patients interact with the health system, which can in turn facilitate action. However, only data directly relevant to taking action should be presented in order to aid information prioritisation. Line charts should be used to display patients’ physiological data where possible to facilitate interpretation and prioritisation. This is supported by evaluations of patient-level dashboards [80], and graphical representations of patient-reported outcome measures (PROMs) [89]. To accommodate user control and freedom [35], maintain appropriate representational formats [27], and aid interpretation [33], users should have the option to also display data as tables. This is supported by EHR design guidelines [90], and studies of PROM [91] and laboratory data [92]. To facilitate perceptual grouping and relationships between data [27], patient-level information should be displayed on a single page. This in turn helps prioritise which clinical areas require most urgent attention, and reduce cognitive overload [26]. Visualisation techniques may help by efficiently summarising patient-level information [93]; LifeLines display data as multiple charts over a common timeline [94]. To provide visually distinct screens for confusable items [27], detailed patient-level information should be completely separate from other interface components. Although this may counter-intuitively interfere with anticipated workflows, it is supported by EHR design guidelines [85]. There should be a warning that the system does not replicate patients’ entire EHRs to avoid false impressions of its completeness and reduce the risk of patient safety events (e.g. a limited medication list may risk drug-drug interactions if new ones are prescribed). Users should have the ability to validate the accuracy of patient-level information, though to fit their workflow only highlighting inconsistencies should be necessary [27].

#### 4.1.4 Suggested actions

Suggested actions may be derived from clinical guidelines, and the wider quality improvement literature. They should align with local clinical pathways, and address specific reasons for users’ under-performance based on detailed analysis of their patients’ EHR data [48], [49]. Suggestions should strive to present ideas users have previously considered, and where possible adapt to contextual features of organisations (e.g. whether they have already been implemented) and individual patients (e.g. clinical contraindications) [51]. This mirrors CDS design guidelines that specify patient-level alerts should incorporate relevant data into decision logic to improve specificity [51]. Suggested actions should: be written concisely (e.g. using bullet points); provide further detail on-demand regarding why it was suggested (i.e. algorithm logic) [51], [56]; report how other organisations achieved change (case reports); and include patient-facing information (e.g. information leaflets or decision aids). They should account for the first step a user may take during implementation such as consulting a patient or discussing a potential organisational change with colleagues. This could be achieved through: the phrasing of the suggested action text (e.g. using goal-setting theory [95]); encouraging users to add their own actions to reflect this workflow; or providing an option to defer their decision about an action [52], [96]. Suggested actions should be displayed to align with user workflow, which may be influenced by whether they are presented on the left or right of the page [82], [97]. They should be prioritised, displaying only the most important three or four options across all quality indicators concurrently, with the option to view more if desired. Preferred prioritisation criteria may vary between users, and should be accommodated with user preference settings where possible. Examples may include: patient safety issues [47], [51]; potential effectiveness; financial value; and quick wins. Prioritisation may be communicated through action ordering or colour [27]. To improve acceptance, they should be presented as advisory [51], [57], and allow users to indicate disagreement (which in turn should be captured and used to improve systems’ algorithms [47], [51]). This may be re-enforced by a warning that suggestions do not over-rule clinical judgment. Users should be able to add their own actions, which should be saved automatically [27], in addition to being used to optimise systems’ own suggestions. Users should be able to save suggested actions, mark them implemented, and view other users’ actions to facilitate intra-organisational teamwork. Saved actions should be displayed at the end of the navigation menu to align with user workflows [47]. It should be possible to order and manually prioritise saved actions, with the facility to set deadlines and reminders, assign them to colleagues, and export them for wider sharing. Response options to suggested actions should be clear [27], and to reduce cognitive load e-A&F systems should automatically detect when an action has been implemented. Where possible, e-A&F systems should facilitate action implementation, which may be helped by integration with existing health information systems [51] such as EHR write-in functionality or direct communication with patients via text message.

### 4.2 Implications for patient safety

Considering our design recommendations (Box 3) in the wider context of A&F causal pathways [15], we can frame their implications on patient safety through three main concepts: user engagement, actionability, and information prioritisation.

User engagement may be influenced through system compatibility, relative advantage, and satisfaction. Compatibility may refer to: the clinical areas addressed by the system; existing systems, policies and workflows with which they align; and user preferences. Ensuring e-A&F systems address clinical areas users deem important and relevant, and that align with existing quality or financial incentive programmes, is essential to ensuring compatibility with their goals and motivation [98]. Similarly, e-A&F systems should align with user workflows, and integrate with existing information systems. Compatibility with user preferences can be improved by enabling user-controlled customisation and tailoring [26], [35]. Incompatible e-A&F systems may not be used by health professionals [99]) or may be dismissed as trivial [100]. PINGR’s relative advantages relate to its provision of detailed patient-level information, suggested actions, and user-friendliness. Ways in which other e-A&F systems can provide relative advantages depends on the individual system and environment into which it is implemented [21]. If a system has a perceived relative advantage, it is more likely to be used and implemented [98]. User satisfaction with e-A&F systems can be influenced by its efficiency and tendency to induce user errors [101]. e-A&F that are more satisfying and positive to use encourage further engagement [102]. Where there is non-engagement with an e-A&F system – whether due to incompatibility, relative disadvantage, or dissatisfaction – potentially important clinical performance information is ignored, which could lead to failures in patient safety (e.g. [6]), whereas continued engagement generally leads to improved patient care [2].

Users must take action based on the information from e-A&F systems in order for improvements in patient care and safety to take place [37]. However, health professionals often do not have the time or skills to translate clinical performance information into improvement action [40]. Therefore maximising the actionability of e-A&F increases this likelihood [2]. Our design recommendations suggest this can be achieved by providing: additional clinical performance data analysis and visualisations; suggested actions; patient lists; and detailed patient-level data. Clinical performance data analyses (such as the improvement opportunities charts in PINGR) can help users understand potential reasons for low performance, and identify groups of patients requiring similar actions [102]. Suggested actions can help users formulate plans for improvement [103]. This is especially true for organisation-level actions, which may have the greatest effect on patient care but clinicians often struggle with most [40], [101]. Optimising suggested action algorithms through user feedback shares best practice between organisations [103] and harnesses positive deviance [104]. Patient lists and detailed patient-level data direct the user’s attention to patients requiring action or further investigation [43]. Not providing patient lists makes it difficult for users to understand who to target or how to improve [40]. Similarly, an absence of detailed patient-level information may mean users fail to take action [101].

Our design recommendations highlight the importance of information prioritisation in all key e-A&F interface components. Health professionals have limited time to dedicate to quality improvement due to competing clinical and non-clinical responsibilities [40], [105]. If their attention is not directed to the most important feedback information, the most appropriate improvement action will likely not occur [19]. For example: focusing on a quality indicator that is not the worst performing may not result in improvement action or the greatest population health gain [106]; reviewing a patient violating multiple quality standards would be more effective than one that violates only one [40]; being unable to view the most important areas of a patient’s information may miss additional, more important areas requiring attention [101]; and implementing an evidence-based action may be more effective than one that is not [98]. An additional benefit of information prioritisation is that it prevents users feeling overwhelmed and disillusioned by the amount of work they perceive needs to be undertaken. Similar to alert fatigue [107], if this happens users may reject the e-A&F system or abandon improvement work altogether [19]. Nevertheless, prioritisation techniques should be used with caution as they may have unintended consequences: for example, using average peer performance as a target may not comprehensively raise standards, as by definition around half the group will already have achieved it.

### 4.3 Study limitations

The team who designed and built the system conducted this evaluation, the lead investigator of which held a position recognisable to study participants as a primary care physician. This posed risks to: the trustworthiness of our findings including how participants behaved [65]; our interpretation of this behaviour [70]; and our degree of positivity in communicating our results [71]. We took specific steps to address these potential problems (Section 2.5 above), and in doing so believe we present a balanced and detailed critique of PINGR. On reflection, author BB found his position may have afforded him insider status [108], gaining more honest insights from participants than a non-medically qualified researcher could elicit. The study’s small sample size (n = 7 participants) may be perceived as a further weakness, though was guided by the achievement of thematic saturation of usability issues [61]. This implies the cost of including further participants would have been unnecessary [59], [60]. Interview transcripts were coded by one researcher, which may be viewed as a threat to credibility [109]. However, we took explicit steps to mitigate this by triangulating findings from multiple data sources [110], in addition to holding critical analytic discussions between authors to challenge any potential biases or assumptions [72]. Our interface design recommendations for e-A&F systems (Box 3) have been derived from empirical studies of one system (PINGR). Although PINGR’s design has been informed by relevant existing usability [3] and theoretical [15], [16] evidence, and its evaluations contextualised in the wider usability literature, effective alternative e-A&F designs may exist. Given the paucity of evidence on e-A&F usability, our recommendations are a reasonable starting point, though should continue to be tested and refined in future. Finally, given this study focused on interface usability in a controlled laboratory setting, it does not address issues that may only be revealed when e-A&F systems are studied in more naturalistic settings [5]. Examples include problems with system implementation, such as how work arising from e-A&F is distributed between health professionals [101], in addition to wider cultural issues, such as whether clinicians are comfortable with scrutiny of their performance by a machine [111].

## 5 Conclusions

We used a combination of qualitative and quantitative methods to evaluate the usability of a novel e-A&F system with target end-users. In doing so, we gained important insights into how to design user-friendly e-A&F systems according to their four key interface components (clinical performance summaries, patient lists, detailed patient-level information, and suggested actions), and how they can be integrated. This enabled us to refine key design recommendations (Box 3) [3], and determine their implications for patient safety. Although our study focused on primary care and long-term conditions, our findings may generalise to other clinical areas and settings.

Each of our data sources (user observation, questionnaires, and interviews) uncovered novel usability issues as well as providing further understanding of those reported by others [10]. Methodologically, we showed how to maximise the discovery of usability issues in a complex health information system [11], and increase the validity of our findings [110], [72]. As far as we are aware, this is the first published study of an e-A&F system to use eye tracking, and therefore presents unique insights into visual search behaviour, integrated within a wider data set.

We report answers to previously identified research questions for e-A&F system design [3], on how to: display information in patient lists; summarise patient-level data across multiple clinical areas; and whether to incorporate clinical performance of other users. This study raises further questions (Box 3); notably how best to prioritise and communicate clinical performance summaries, patient lists, and suggested actions – a grand challenge of CDS [29] – and how these findings translate to more naturalistic settings. Future work will seek to address these questions in further phases of our iterative development framework [5]. Finally, our findings provide further support for, and an example of, multi-method approaches in usability studies [12], [13].

## Author contributions

All authors contributed to the conception and design of the study. BB conceived and designed PINGR, which was built by both RW and BB. BB collected the data. All authors analysed the data. All authors contributed to and approved the final version of the manuscript.

## Funding

This work was supported by a Wellcome Trust Research Training Fellowship for BB [104438/Z/14/Z]; the MRC Health e-Research Centre, Farr Institute of Health Informatics Research [MR/K006665/1]; and the National Institute for Health Research Greater Manchester Primary Care Patient Safety Translational Research Centre (NIHR Greater Manchester PSTRC). The views expressed are those of the author(s) and not necessarily those of the NHS, the NIHR or the Department of Health.

## Conflict of interest

None.
