# Supplementary material for: Multi-method laboratory user evaluation of an actionable clinical performance information system: Implications for usability and patient safety
Source: J Biomed Inform. 2018 Jan;77:62–80. doi: 10.1016/j.jbi.2017.11.008 (PMC5766660; doi:10.1016/j.jbi.2017.11.008)
Supplement: Supplementary data 1 [file mmc1.docx]

**Appendix A: Screening Questionnaire**

1. **Personal characteristics**

| **Participant ID [to be completed by researcher]** |  |
| --- | --- |
| **Today’s date [to be completed by researcher]** |  |
| **Gender** | MALE / FEMALE |
| **Age** | 15-24 / 25-34 / 35-44 / 45-54 / 55-64 / 65+ |
| **Current job** |  |
| **Year started current job** |  |
| **Years of experience undertaking audit / QI** |  |

1. **Familiarity with Computers and the Web**

**How frequently do you use computers and the World Wide Web (WWW) during the week** (please circle)

| **Desktop computers or laptops:**  E.g. MS Word office, Internet, other client applications | 1 | 2 | 3 | 4 |
| --- | --- | --- | --- | --- |
| **World Wide Web:**  E.g. Search engines, Social networking sites, other websites | 1 | 2 | 3 | 4 |

[1 = Less than an hour per week; 2 = One to four hours per week; 3 = Five to 10 hours per week; 4 = More than 10 hours per week]:

1. **Use of health information systems and applications**

**On the days in which you work in your current job**, how often do you use the following types of software (please circle):

|  | Never |  | Half the days |  | Every day |
| --- | --- | --- | --- | --- | --- |
| **Electronic Health Records**  E.g. EMIS, Vision, SystmOne | 1 | 2 | 3 | 4 | 5 |
| **Audit software**  E.g. QOF reporting tools, ‘Population reporting’ function in EMIS, BMJ Informatica Audit+, PRIMIS GRASP-AF, IMPAKT CKD tool | 1 | 2 | 3 | 4 | 5 |
| **“Pop-ups”, “reminders” and “alerts” within Electronic Health Records**  E.g. Medication alerts, QOF pop-ups “Pop-ups” and alerts within Electronic Health Records, templates within Electronic Health Records | 1 | 2 | 3 | 4 | 5 |
| **Information retrieval applications**  E.g. Websites like ‘Map of Medicine’ or ‘CKS’, Information-buttons like ‘Web Mentor’ within EMIS. | 1 | 2 | 3 | 4 | 5 |
| **Clinical risk calculators**  E.g. iPhone apps for Well’s scores or QRISK website | 1 | 2 | 3 | 4 | 5 |
| **Documentation templates**  E.g. Standardised templates within Electronic Health Records | 1 | 2 | 3 | 4 | 5 |
| **Decision support systems**  E.g. Diagnostic support tools like ‘Isabel’ | 1 | 2 | 3 | 4 | 5 |
