# Supplementary material for: Multi-method laboratory user evaluation of an actionable clinical performance information system: Implications for usability and patient safety
Source: J Biomed Inform. 2018 Jan;77:62–80. doi: 10.1016/j.jbi.2017.11.008 (PMC5766660; doi:10.1016/j.jbi.2017.11.008)
Supplement: Supplementary data 2 [file mmc2.docx]

**Appendix B: Tasks**

All participants were provided with the same contextual background information about a fictional primary care practice each time they were given a task. They were asked to use this information to inform their judgments during the tasks.

“You are a GP partner at Grove Medical Practice, located in a small industrial town in North West England. You have just over 10000 patients registered who are mainly elderly with high rates of multimorbidity. There are 6 full-time partners (including you) and one part-time salaried doctor. You employ 3 nurses, 1 health-care assistant, 1 practice manager, 1 deputy practice manager, and 10 other administrative and reception staff. Historically you have been a high-achieving QOF practice, but recent changes to your contract mean your income has dropped significantly in the last 2 years. You have therefore agreed with your partners that only changes to the practice that are cost-neutral, cost-saving or significantly improve patient care should be implemented.”

**Task 1:**

You want to make improvements at a ***practice-level*** for patients with ***atrial fibrillation*** at your practice.

Please use the software find relevant suggested ***improvement actions***, and indicate ***whether or not you agree*** with them given the information presented.

**Task 2:**

You want to improve the management of a ***patient*** with ***uncontrolled hypertension according to NICE targets***.

Choose the patient you think it would be ***most important to address first*** according to your own judgment (you are free to use whatever criteria you wish – there is no right answer). Then find the suggested ***improvement actions*** for this patient and indicate ***whether or not you agree*** with them given the information in the software and the patient’s medical record (below).

**Extract of medical record**

(This information applies to whichever patient you choose to look at)

**Sex:** Female **Age:** 71

**Problems:** Hypertension **Medication:** Amlodipine 10mg OD

**Allergies:** ACE-inhibitors (cough)

**Latest consultation:** Refuses to have home blood pressure monitoring

**Task 3:**

You are about to see ***patient 123*** in surgery. Please find whether there are any suggested ***improvement actions*** that could be used to improve their care, and if yes, indicate ***whether or not you agree*** with them given the information in the software and the patient’s medical record (below).

**Extract of medical record**

**Sex:** Male **Age:** 65

**Problems:** Hypertension, atrial fibrillation, chronic kidney disease

**Medication:** Warfarin 1mg as directed, Aspirin 75mg OD, Amlodipine 10mg OD, Ramipril 10mg OD, Bisoprolol 5mg OD, Indapamide 2.5 mg OD,

**Allergies:** Nil known

**Task 4:**

You have an idea to improve how your ***practice*** cares for patients with ***asthma***.

Your idea is to ***invite a local consultant to do a talk***.

Please add this to the software where you judge is most appropriate.

**Task 5:**

Please find the ***patient with the most improvement opportunities available*** at your practice ***across all conditions***.

**Task 6:**

You want to implement all the improvement **actions** you have agreed with in the software that are ***awaiting implementation*** (both at practice-level and patient-level).

Please ***download*** them so you can share them with your colleagues at your practice and ask for their help to implement them.

**Task 7:**

You have now ***implemented*** all of the improvement actions you downloaded in the previous activity.

Please ***choose one*** (whichever you like, there is no right answer) and ***indicate in the software that it has been implemented***.
