# Supplementary material for: Multi-method laboratory user evaluation of an actionable clinical performance information system: Implications for usability and patient safety
Source: J Biomed Inform. 2018 Jan;77:62–80. doi: 10.1016/j.jbi.2017.11.008 (PMC5766660; doi:10.1016/j.jbi.2017.11.008)
Supplement: Supplementary data 3 [file mmc3.docx]

**Appendix C: Post-test questionnaires**

**System Usability Scale (SUS)** [1]

|  | Strongly disagree |  |  |  | Strongly agree |
| --- | --- | --- | --- | --- | --- |
| I think that I would like to use this system frequently | 1 | 2 | 3 | 4 | 5 |
| I found the system unnecessarily complex | 1 | 2 | 3 | 4 | 5 |
| I thought the system was easy to use | 1 | 2 | 3 | 4 | 5 |
| I think that I would need the support of a technical person to be able to use this system | 1 | 2 | 3 | 4 | 5 |
| I found the various functions in this system were well integrated | 1 | 2 | 3 | 4 | 5 |
| I thought there was too much inconsistency in this system | 1 | 2 | 3 | 4 | 5 |
| I would imagine that most people would learn to use this system very quickly | 1 | 2 | 3 | 4 | 5 |
| I found the system very cumbersome to use | 1 | 2 | 3 | 4 | 5 |
| I felt very confident using the system | 1 | 2 | 3 | 4 | 5 |
| I needed to learn a lot of things before I could get going with this system | 1 | 2 | 3 | 4 | 5 |

**References**

[1] J. Brooke, SUS: A “quick and dirty” usability scale, in: Usability Eval. Ind., Taylor and Francis)., London, 1996.

**Action Interface questionnaire**

Participant ID:

| Actions^[[1]](#footnote-1)^ | Difficult |  |  |  | Easy |
| --- | --- | --- | --- | --- | --- |
| 1. Agreeing and disagreeing with practice-level actions for atrial fibrillation was… | 1 | 2 | 3 | 4 | 5 |
| 2. Agreeing and disagreeing with actions for a patient with uncontrolled hypertension was… | 1 | 2 | 3 | 4 | 5 |
| 3. Agreeing and disagreeing with actions for patient 123 was… | 1 | 2 | 3 | 4 | 5 |
| 4. Adding an action to invite a consultant to do a talk was… | 1 | 2 | 3 | 4 | 5 |
| 5. Finding the patient with the most improvement opportunities was… | 1 | 2 | 3 | 4 | 5 |
| 6. Downloading your plan was… | 1 | 2 | 3 | 4 | 5 |
| 7. Indicating an action had been implemented was… | 1 | 2 | 3 | 4 | 5 |

**Object interface questionnaire**

|  | Unclear |  |  |  | Clear |
| --- | --- | --- | --- | --- | --- |
| 8. The presentation of practice-level performance data was… | 1 | 2 | 3 | 4 | 5 |
| 9. The presentation of patient lists was… | 1 | 2 | 3 | 4 | 5 |
| 10, The presentation of patient-level data was… | 1 | 2 | 3 | 4 | 5 |
| 11. The presentation of action plans was… | 1 | 2 | 3 | 4 | 5 |
| 12. The use of charts was… | 1 | 2 | 3 | 4 | 5 |
| 13. The language used in the application was… | 1 | 2 | 3 | 4 | 5 |
| 14. The use of colour in the application was… | 1 | 2 | 3 | 4 | 5 |
| 15. The font used was… | 1 | 2 | 3 | 4 | 5 |

1. A full description of each task is detailed in Table 1 of the manuscript [↑](#footnote-ref-1)
